# The Association Between Brain Metabolic Biomarkers Using ^18^F-FDG and Cognition and Vascular Risk Factors, as well as Its Usefulness in the Diagnosis and Staging of Alzheimer’s Disease

**DOI:** 10.3233/ADR-240104

**Published:** 2024-09-05

**Authors:** Min Xiong, Hongji You, Wang Liao, Yingren Mai, Xiaoming Luo, Yipei Liu, Sheng-nan Jiang

**Affiliations:** a Department of Nuclear Medicine, the Second Affiliated Hospital, Guangzhou Medical University, Guangzhou, China; b Department of Neurology, the Second Affiliated Hospital, Guangzhou Medical University, Guangzhou, China

**Keywords:** Alzheimer’s disease, cognitive function, ^18^Fluorodeoxyglucose, metabolism, mild cognitive impairment, positron emission tomography, risk factor

## Abstract

**Background:**

^18^F-fluorodeoxyglucose (^18^F-FDG) positron emission tomography (PET) is valuable in Alzheimer’s disease (AD) workup.

**Objective:**

To explore the effectiveness of ^18^F-FDG PET in differentiating and staging AD and associations between brain glucose metabolism and cognitive functions and vascular risk factors.

**Methods:**

107 participates including 19 mild cognitive impairment (MCI), 38 mild AD, 24 moderate AD, 15 moderate-severe AD, and 11 frontotemporal dementia (FTD) were enrolled. Visual and voxel-based analysis procedures were utilized. Cognitive conditions, including 6 cognitive function scores and 7 single-domain cognitive performances, and vascular risk factors linked to hypertension, hyperlipidemia, diabetes, and obesity were correlated with glucose metabolism in AD dementia using age as a covariate.

**Results:**

^18^F-FDG PET effectively differentiated AD from FTD and also differentiated MCI from AD subtypes with significantly different hypometabolism (except for mild AD) (height threshold *p* < 0.001, all *puncorr* < 0.05, the same below). The cognitive function scores, notably Mini-Mental State Examination and Montreal Cognitive Assessment, correlated significantly with regional glucose metabolism in AD participants (all *p* < 0.05), whereas the single-domain cognitive performance and vascular risk factors were significantly associated with regional glucose metabolism in MCI patients (all *p* < 0.05).

**Conclusions:**

This study underlines the vital role of ^18^F-FDG PET in identifying and staging AD. Brain glucose metabolism is associated with cognitive status in AD dementia and vascular risk factors in MCI, indicating that ^18^F-FDG PET might be promising for predicting cognitive decline and serve as a visual framework for investigating underlying mechanism of vascular risk factors influencing the conversion from MCI to AD.

## INTRODUCTION

Dementia is an umbrella term encompassing a range of clinical syndromes and is among the leading causes of death in the elderly population.^1^ The etiology and pathological manifestations of dementia are varied, with primary types including frontotemporal dementia (FTD), Lewy body dementia (LBD), and Alzheimer’s disease (AD). AD is a complex neurodegenerative disorder characterized by progressive cognitive decline and memory loss, and it is the most prevalent subtype of dementia. In AD patients, three primary types of neuropathological changes emerge: extracellular neuritic plaques composed of amyloid-β (Aβ), intracellular neurofibrillary tangles made up of hyperphosphorylated tau protein, and neuroinflammation,^2,3^ followed by the pattern of synaptic dysfunction and neuronal loss. It represents a spectrum from cognitive normal to mild cognitive impairment (MCI) and eventually to AD.^4^ AD is categorized into three stages of progression based on disease severity: mild, moderate, and severe. Differentiating MCI from AD subtypes is crucial for disease treatment and management, which seems to be a difficult task for Aβ positron emission tomography (PET)-based molecular imaging.^5^ FTD is the second cause of early-onset dementia, marked by progressive mental behavior abnormalities and dysfunctions in executive or language abilities. The pathological hallmarks of FTD include gliosis, micro-vacuolation, synaptic and neuronal loss, and the presence of specific molecular protein aggregates like tau.^6^ Thus, symptoms and tau pathology overlaps exist in two dementia subtypes, posing a challenge for clinical identification and management.^7^

Molecular imaging focuses on the fundamental changes in biological processes, enabling the detection of diseases in pre-symptomatic phases. Molecular neuroimaging with ^18^Fluorodeoxyglucose (^18^F-FDG), which accumulates around synapses and reflects local neuronal activity, holds promise for disease diagnosis and visualization of underlying pathology. ^18^F-FDG PET has been shown to reveal distinct hypometabolic patterns in two types of dementia, and its use has been endorsed by various guidelines for the comprehensive workup of dementia.^8^ The reduction in glucose metabolism is indicative not only of the severity of neuronal dysfunction, which aids in the staging of AD, but also helps to understand the potential connections between brain function and patient behaviors and symptoms.^9^ Previous studies using ^18^F-FDG PET have demonstrated that glucose uptake in specific brain regions of AD patients correlates positively with cognitive score on the Mini-Mental State Examination (MMSE) and performance on the semantic verbal fluency test, which assesses language ability and executive functions.^10,11^ There is increasing concern that AD can co-occur with a series of chronic conditions, such as diabetes, hypertension, hypercholesterolemia, and obesity, which are considered vascular risk factors and may influence the risk of developing AD.^12,13^ Earlier research suggested that AD shares numerous cellular and molecular pathological pathways with diabetes and hypertension.^12,14^ Animal-based studies have provided evidence that vascular risk factors can exacerbate Aβ deposition in AD following diet-induced conditions like diabetes, hypertension, or hypercholesterolemia.^14–16^ Furthermore, the association between the physical state of the elderly and cognitive dysfunction has attracted public attention, with an increased body mass index (BMI) potentially indicating cognitive dysfunction in FTD.^17^ However, it is still unclear whether there is a relationship between vascular risk factors and brain glucose metabolism in AD dementia and which of them worsens the fundamental neuropathological changes.

Therefore, we performed ^18^F-FDG PET/CT imaging in MCI, AD, and FTD individuals from a single-center and case-control study to verify the ability of ^18^F-FDG PET/CT to distinguish continuous spectrum of AD from MCI and FTD. Additionally, we aim to investigate the association between cerebral glucose metabolism in AD dementia and cognitive function, as well as the impact of existing vascular risk factors on this process.

## MATERIALS AND METHODS

### Participants

**Fig. 1:**
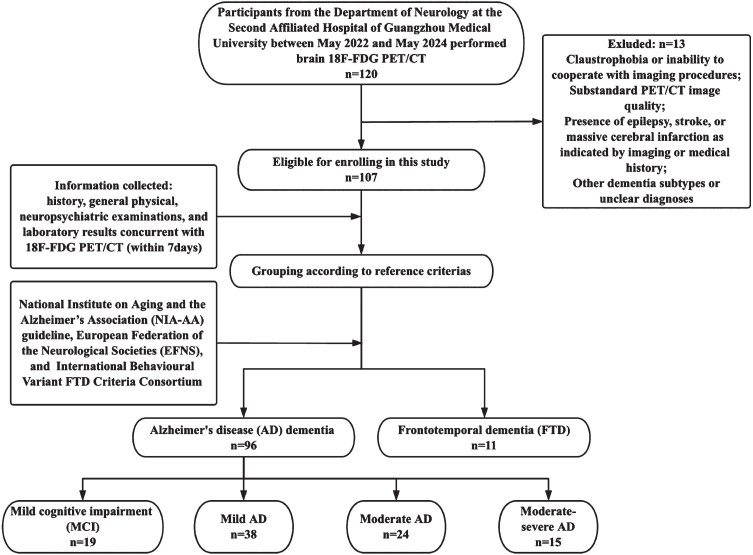
Overview of the study design and inclusion of participants.

Patients diagnosed with MCI, AD, or FTD at the Department of Neurology, the Second Affiliated Hospital, Guangzhou Medical University, from May 2022 to May 2024, and who underwent ^18^F-FDG PET/CT in the Department of Nuclear Medicine, were recruited for the study. The study cohort comprised 107 subjects: 19 with MCI, 38 with mild AD, 24 with moderate AD, 15 with moderate-severe AD, and 11 with FTD. Concurrently, MCI and AD patients received Aβ deposition (*n* = 93) (^18^F-florbetapir, ^18^F-AV45) and tau protein (*n* = 96) (^18^F-flortaucipir, ^18^F-AV1451) assessments to meet the dementia criteria set by the National Institute on Aging and the Alzheimer’s Association (NIA-AA) guidelines.^18,19^ FTD diagnoses were based on the European Federation of the Neurological Societies (EFNS) and International Behavioural Variant FTD Criteria Consortium.^20,21^ The exclusion criteria included: 1) Allergy to the imaging tracer or presence of contraindications for the tracer injection, such as severe liver or kidney failure; 2) Claustrophobia or inability to cooperate with imaging procedures; 3) Substandard PET/CT image quality; 4) Presence of epilepsy, stroke, or massive cerebral infarction as indicated by imaging or medical history; and 5) Other dementia subtypes or unclear diagnoses. A schematic of the study design and inclusion strategy is depicted in Fig. 1. This study was approved by the Ethics Committee of the Second Affiliated Hospital, Guangzhou Medical University (Approval No. 2023-HG-KS-46) and was conducted in accordance with the Helsinki Declaration of 1975. Informed consent was obtained from all patients.

### Neuropsychological and clinical assessment

A comprehensive history, general physical, and neuropsychiatric examinations were conducted by neurologists specializing in dementia care. Demographic information including age, gender, and education level was documented. The comprehensive cognitive scales integral to diagnosing and differentiating AD were utilized, including the MMSE, Montreal Cognitive Assessment (MoCA), Activity of Daily Living Scale (ADL), Gesell Developmental Schedule (GDS), Hachinski Ischemic Score (HIS), and Hamilton Anxiety Scale (HAMA). According to the neurological examination, single-domain cognitive assessments encompassing learning, calculation, orientation, visual-spatial skill, hallucination, personality, and emotional status were performed and categorized as normal or declined/abnormal. Given the inaccurate time of chronic illnesses (diabetes, hypertension, obesity, and hypercholesterolemia) caused by the confused memories of patients, the study focused on objective serological markers and BMI as indicators of vascular risk factors. To be specific, laboratory data, obtained within a week of ^18^F-FDG PET/CT, included glycosylated hemoglobin (HbA1c), total cholesterol (TC), low-density lipoprotein (LDL), high-density lipoprotein (HDL), triglycerides (TG), homocysteine (Hcy), folate, and vitamin B12 (VB12). BMI was calculated using weight (kg) divided by the square of height (m). Smoking history was minimal among participants and thus not considered a risk factor in this analysis. Brain MRI was obtained from patients who had completed the examination.

### PET acquisition and preprocessing

For the ^18^F-FDG PET/CT examination, all subjects were instructed to fast for at least 6 hours and to maintain their blood glucose levels below 11.1 mmol/L. Brain PET/CT imaging (GE Discovery 710, USA) was acquired 60–80 min after the intravenous injection of ^18^F-FDG (injection dose 3.7–5.55 MBq/kg, radiochemical purity > 95%) with subject in a dimly lit room and eyes closed to minimize auditory stimulation. The acquisition time was 10 min. For the ^18^F-AV45 and ^18^F-AV1451 examinations, no additional preparation was needed, with an injection dose of 370 MBq/person and an acquisition time of 10–15 min. The detailed image acquisition procedure was as follows: Subjects were positioned supine, and their heads were fixed in a special headrest with the scanning range from the top of the skull to the base. Initially, a low-dose CT scan (voltage: 120 KV, current: 100–180 mA, slice thickness: 3.75 mm) was proceeded for PET attenuation correction, followed by 3D brain PET acquisition. The ordered subset expectation maximization (OSEM) algorithm was utilized for image reconstruction, with a reconstruction matrix of 256×256×256 and voxel dimensions of 2.73 mm×2.73 mm×2.79 mm. The workstation (AW VolumeShare 7, GE) was applied to exhibit PET, CT, and PET/CT cross-sectional, coronal, and sagittal images, as well as PET maximal intensity projection (MIP) images. Finally, all PET/CT scans were assessed and reviewed by nuclear medicine physicians who specialize in neuroimaging to ensure quality control.

### PET images processing, analysis, and statistics

*Visual assessment of PET/CT images.* All images were visually assessed to yield dichotomous results by two experienced nuclear medicine physicians. Any disagreements were resolved through discussion and consensus. For AD dementia, a positive diagnosis was indicated by reduced ^18^F-FDG metabolism in the gray matter of the bilateral parietal and temporal lobes, as well as the posterior cingulate and precuneus gyri. The hypometabolism in the frontal and anterior temporal cortices was defined as FTD positive.

*SPM data processing.* PET images were preprocessed using Statistical Parametric Mapping 12 (SPM12; Institute of Neurology, University College of London, UK) implemented on the MATLAB R2012a platform (MathWorks Inc, Natick, MA, USA). The initial step involved converting raw DICOM data into a format compatible with further analysis using SPM12. Subsequently, the converted images were spatially standardized according to the Montreal Neurological Institute (MNI) brain atlas to obtain normalized anatomical spatial images.^22^ Finally, images were smoothed with 10 mm×10 mm×10 mm full width half maximum (FWHM) to improve the image signal-to-noise ratio (SNR) and generate target images.

*Data statistics analysis.* Statistical analysis was conducted using SPSS Statistics version 26 (IBM, Armonk, NY, USA) and SPM12. Continuous variables were described using mean (standard deviation, SD) or median (interquartile range, IQR), while categorical variables were expressed as frequency or percentage. In terms of the metabolic distribution among different groups, the SPM two-sample *t*-test statistic was employed, and the spatial coordinates of the pixels with statistical significance were obtained. The Automated Anatomical Labeling (AAL) template dividing the brain into 116 sub-regions was subsequently applied to identify the functional brain regions corresponding to the abnormal pixels. Using age as a covariate, we assessed the correlation between cerebral metabolism and cognitive function scores, single-domain cognitive measures, and existing vascular risk factors in AD dementia through multiple regression models of SPM12. The statistical significance of SPM12 was set at a height threshold of *p* < 0.001 with the familywise error rate (FWE) uncorrected and *puncorr* < 0.05 (Later referred to as *p*) at the cluster-level, excluding clusters smaller than 3 times expected voxels. The statistical significance of SPSS was set at *p* < 0.05.

## RESULTS

### Demographic and clinical characteristics

A total of 107 participants were enrolled in this study, comprising 19 individuals with MCI (men 5, women 14; mean age 67.42±8.06), 77 with AD (men 23, women 73; mean age 67.94±8.42 years; 38 with mild AD, 24 with moderate AD, 15 with moderate-severe AD), and 11 with FTD (man 1, women 10; median age 62(60,66)). No statistically significant differences were found in age and gender among the three disease classes. Table 1 summarizes the demographic and clinical characteristics of MCI and AD subtypes, as well as the statistical results for comparisons between groups. Significant differences were found in MMSE and MoCA scores across the four groups, with scores decreasing as the increased severity of the disease (both *p* < 0.001). Furthermore, the higher the stage of the disease, the worse the ability to perform daily living (*p* < 0.001).

**Table 1:** Clinical and demographic data of MCI and AD patients

Characteristics	MCI group	Mild AD group	Moderate AD group	Moderate-severe	*p*
	Median (IQR)	Median (IQR)	Median (IQR)	AD group
				Median (IQR)
Age	66 (60,74)	71.5 (65,75.25)	66 (59.25,77)	66 (56,70)	0.054
Gender (Women)	14 (73.68%)	29 (75.32%)	19 (79.17%)	11 (73.33%)	0.969
Education (y)	12 (6.75,12)	9 (0,12)	6 (6,12)	6 (0,15)	0.194
MMSE	25 (21.75,27.25)	20 (15.5,23)	11 (11,21)	5 (1,15)	<0.001^*^
MoCA	18.5 (15.5,25.5)	15 (10,17.5)	9 (6,15)	4 (1.5,11)	<0.001^*^
ADL	14 (14,14.25)	14 (14,18.5)	16 (14,23)	30.5 (18.75,50.5)	<0.001^*^
GDS	11 (7.25,13)	5 (2,7.5)	5 (4,14)	7 (4,10.25)	0.112
HIS	1 (1,2)	1 (1,2.5)	3 (2,5)	2 (1,2.75)	0.048^*^
HAMA	5 (3,9.25)	3 (2,5)	6 (4,10)	4.5 (3,9.25)	0.001^*^
BMI	21.85 (19.16,22.97)	22.76 (21.35,24.67)	22.83 (21.05,25.78)	22.32 (20.11,27)	0.656
HbA1c	5.6 (5.43,5.95)	5.95 (5.5,6.45)	5.7 (5.4,6)	5.5 (5.4,6.1)	0.243
TC	4.69 (3.8,4.88)	4.86 (3.65,5.29)	5.44 (4.19,5.66)	4.83 (4.13,5.23)	0.255
LDL	2.95±1.03^a^	3.06±0.82^a^	3.46±0.79^a^	3.17±1.2^a^	0.552
HDL	1.28 (1.08,1.5)	1.21 (1.06,1.43)	1.2 (1.09,1.33)	1.16 (0.7,1.32)	0.382
TG	1.28 (0.57,1.73)	1.02 (0.93,1.3)	1.23 (1.07,1.68)	1.35 (1.2,1.74)	0.080
Hcy	10.74 (9.03,13.31)	12.98 (8.76,15.67)	9.34 (8.75,12.58)	9.98 (8.68,13.06)	0.588
Folate	11.8 (10.3,22.8)	17.2 (12.3,28.05)	15.7 (13.3,23.85)	9.2 (8.4,13.7)	0.005^*^
VB12	236 (199,355)	337 (207,481)	297 (254,460.5)	440 (284,555)	0.671

^*^p<0.05, the difference was statistically significant; ^a^Data was presented as mean±SD. AD, Alzheimer’s disease; MCI, mild cognitive impairment; MMSE, Mini-Mental State Examination; MoCA, Montreal Cognitive Assessment; ADL, Activity of Daily Living Scale; GDS, Gesell Developmental Schedule; HIS, Hachinski Ischemic Score; HAMA, Hamilton Anxiety Scale; BMI, Body Mass Index; HbA1c, glycosylated hemoglobin; TC, total cholesterol; LDL, low-density lipoprotein; HDL, high-density lipoprotein; TG, triglycerides; Hcy, homocysteine; VB12, vitamin B12.

### Comparison of distribution of glucose load in subjects with different AD subtypes, MCI, and FTD

We evaluated the diagnostic performance of visual assessment using ^18^F-FDG PET for identifying AD, MCI, and FTD. The positive detection rates were as follows: 47.37% (9/19) for MCI, 55.26% (21/38) for mild AD, 75% (18/24) for moderate AD, 93.33% (14/15) for moderate-severe AD, and 100% (11/11) for FTD patients. Consequently, the diagnostic performance of ^18^F-FDG PET in AD is association with disease status.

**Fig. 2:**
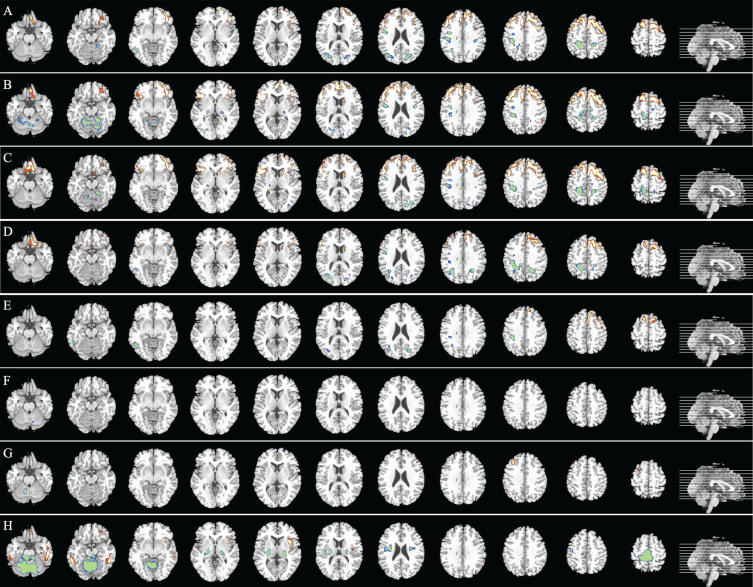
Differential distribution of glucose metabolism between FTD and AD dementia (MCI and three AD subtypes), as well as between MCI and three AD subtypes groups. A) MCI and AD versus FTD: Regions highlighted in red (former) indicate areas of higher metabolism in MCI and AD than in FTD, while green (later) indicates the reverse. This color scheme applies to all subsequent comparisons. B) MCI versus FTD. C) mild AD versus FTD. D) moderate AD versus FTD. E) moderate-severe AD versus FTD. F) MCI versus mild AD. G) MCI versus moderate AD. H) MCI versus moderate-severe AD. FTD, frontotemporal dementia; AD, Alzheimer’s disease; MCI, mild cognitive impairment.

Subsequently, we conducted a quantitative analysis to assess metabolic differences among the groups. Consistently, the FTD group exhibited lower metabolism in the frontal lobe compared to MCI, three AD subtypes, and all AD dementia (all *p* < 0.05; Fig. 2). Conversely, FTD patients (except for those with moderate-severe AD) demonstrated higher metabolism in the parietal lobe compared to MCI, mild AD, and moderate AD patients (all *p* < 0.05; Fig. 2). In comparative analysis between MCI and the three AD subtypes, no significant differences in brain metabolism were uncovered between MCI and mild AD patients (all *p* > 0.05). The glucose metabolism of the frontal lobe (Frontal_mid_2R) in the MCI group was higher than in the moderate AD group (*p* = 0.046). The glucose metabolism of the temporal lobe (Temporal_Inf_L) and insular lobe (Insula_L) in MCI group was higher than in the moderate-severe AD group as well (*p* = 0.009 and 0.027; Fig. 2). Interestingly, the higher uptake in the paracentral lobule (Paracentral_Lobule_R), thalamus (Thal_PuL-L), insular lobe (Insula_R), and cerebellum (Cerebelum_Crusl1_L) in moderate-severe AD group was found compared with the MCI group (all *p* < 0.05).

### Neuropsychological assessment scale associated with brain glucose metabolism of AD and MCI

The relationship between cognitive function scores and glucose metabolism in all AD patients was evaluated (Table 2). As a result, the MMSE score showed a significant positive association with glucose metabolism in the parietal lobe (Parietal_Inf_L) and fusiform gyrus (Fusiform_L) (both *p* < 0.05). In addition, the reduction of ^18^F-FDG uptake in the parietal lobe (Parietal_Inf_L) and temporal lobe (Temporal_Mid_L) was positively correlated with the MoCA score (both *p* < 0.05). On the contrary, the metabolism in the frontal lobe (Frontal_Sup_2_L) and occipital lobe (Occipital_Sup_L) demonstrated a significant negative association with the ADL score (both *p* < 0.05). The ^18^F-FDG accumulation in the postcentral gyrus (Postcentral_R) and hippocampus (Hippocampus_R) was negatively related to the GDS score (both *p* < 0.05). No evidence was found to support the association between cerebral glucose metabolism and scores on the HIS and HAMA (all *p* > 0.05). Subsequently, we analyzed the MCI group and did not find any statistically significant associations similar to those observed in the AD group.

**Table 2:** The statistically significant associations between glucose metabolism and cognitive assessment in AD and MCI groups

Cognitive function scores	AD group		Single-domain cognitive performances	MCI group	
	Positive	Negative	Positive	Negative
MMSE	Fusiform_L	Thal_PuI_R	Learning	Olfactory_L	OFCmed_R
	Parietal_Inf_L	—	Calculation	ParaHippocampal_L	ACC_sub_R
MoCA	Temporal_Mid_L	Rectus_R	Orientation	Olfactory_L	OFCmed_R
	Parietal_Inf_L	—	Visual-spatial skill	Olfactory_L	OFCmed_R
ADL	—	Frontal_Sup_2_L	Personality	Parietal_Inf_L	—
	—	Occipital_Sup_L		Paracentral_Lobule_L	—
GDS	—	Postcentral_R		Cingulate_Mid_L	—
		Hippocampus_R	Emotional status	—	Rolandic_Oper_L
HIS	—	—		—	OFCmed_R
HAMA	—	—	Hallucination	ParaHippocampal_L	—

AD, Alzheimer’s disease; MCI, mild cognitive impairment; MMSE, Mini-Mental State Examination; MoCA, Montreal Cognitive Assessment; ADL, Activity of Daily Living Scale; GDS, Gesell Developmental Schedule; HIS, Hachinski Inchemic Score; HAMA, Hamilton Anxiety Scale. The above results were statistically significant with *p* < 0.05.

### Single-domain cognitive performances involved in AD dementia development associated with brain glucose metabolism

To assess whether the connection between single-domain cognitive functions in AD dementia and neuronal pathological injury is revealed by ^18^F-FDG PET, we estimated the potential associations between glucose metabolism and specific cognitive statuses in patients with AD and MCI who had completed the assessments (Table 2). Significant positive correlations were noted between glucose metabolism in the left olfactory bulb (Olfactory_L) and the abilities of learning, orientation, and visual space in the MCI group (all *p* < 0.05). The skills of learning and visual space in MCI and AD patients, as well as emotional status in MCI patients were negatively associated with ^18^F-FDG uptake in the orbitofrontal cortex (OFCmed_R) (MCI: all *p* < 0.05; AD: both *p* > 0.05). In the MCI group, personality status was found to be positively related to metabolism in the parietal lobe (Parietal_Inf_L), paracentral lobule (Paracentral_Lobule_L), and cingulate gyrus (Cingulate_Mid_L) (all *p* < 0.05). Calculation ability and the presence of hallucination in the MCI group were positively associated with metabolism in the parahippocampal gyrus (ParaHippocampal_L) (both *p* < 0.05). For AD patients, hallucination was related to metabolism in the hippocampal (Hippocampus_L) (*p* = 0.58).

### Existing vascular risk factors associated with brain glucose metabolism of AD dementia

In this section, we explored the impact of existing vascular risk factors on brain metabolism in patients with AD and MCI (Table 3). Among the MCI population, it was exposed that the retention of ^18^F-FDG in the paracentral lobule (Paracentral_Lobule_R) and supplementary motor area (Supp_Motro_Area_L) was positively associated with BMI (both *p* < 0.05). A similar positive association was observed between HbA1c values and metabolism in the parietal lobe (Parietal_Inf_L) (*p* = 0.013). Additionally, there were positive associations between TC levels and metabolism in the occipital lobe (Occipital_Sup_L) and cuneus (Cuneus_R), as well as between LDL levels and metabolism in the occipital lobe (Occiptical_Mid_R and Occiptical_Sup_L) (all *p* < 0.05). However, the levels of HDL were negatively related to the glucose uptake in the calcarine fissure (Calcarine_L) and frontal lobe (Frontal_sup_2_R) (both *p* < 0.05). As for factors like Hcy, Folate, and VB12, we found that higher Hcy level appeared to be linked with lower glucose accumulation in the fusiform gyrus (Fusiform_R) (*p* = 0.017). Inversely, positive correlations were identified between the folate levels and orbitofrontal cortex (OFCant_R) glucose uptake, and between the VB12 values and ^18^F-FDG uptake in the temporal lobe (Temporal_Mid_L) and postcentral gyrus (Postcentral_R) (all *p* < 0.05). Unfortunately, no correlations were uncovered when examining the relationship between serum markers and BMI with brain uptake in the AD populations (all*p* > 0.05).

**Table 3:** The statistically significant associations between glucose metabolism and vascular risk factors in MCI group

	MCI group	
Vascular risk factors	Positive	Negative
BMI	Paracentral_Lobule_R	—
	Supp_Motro_Area_L	—
HbA1c	Parietal_Inf_L	—
TC	Occiptical_Sup_L	—
	Cuneus_R	—
LDL	Occiptical_Mid_R	—
	Occiptical_Sup_L	—
HDL	—	Frontal_sup_2_R
		Calcarine_L
TG	—	—
Hcy	—	Fusiform_R
Folate	OFCant_R	Paracentral_Lobule_R
	—	Precuneus_L
VB12	Temporal_Mid_L	Precuneus_L
	Postcentral_R	Precuneus_R

MCI, mild cognitive impairment; BMI, body mass index; HbA1c, glycosylated hemoglobin; TC, total cholesterol; LDL, low-density lipoprotein; HDL, high-density lipoprotein; TG, triglycerides; Hcy, homocysteine; VB12, vitamin B12. The above results were statistically significant with *p* < 0.05.

## DISCUSSION

^18^F-FDG PET is a pioneering and extensively available neuroimaging technique for diagnosing and differentiating dementia, and it remains one of the most valuable components for AD workup. We investigated the metabolic discrepancies among FTD, MCI, and AD subtypes using ^18^F-FDG PET/CT and explored the association between cognitive functions, existing vascular risk factors, and brain glucose metabolism in AD dementia. We found that distinct hypometabolic regions existed between AD dementia and FTD, as well as between MCI and AD subtypes in quantitative analysis, reinforcing the value of ^18^F-FDG PET as a robust diagnostic and progression assessment tool for AD. Furthermore, comprehensive cognition scores of AD populations were associated with specific regional metabolism, while single-domain cognitive status and vascular-related risk factors were associated with glucose metabolism in discrete brain regions in MCI patients. Thus, this study underscores the potential of ^18^F-FDG PET imaging as a promising approach for identifying disease-affected regions that correspond to neuropsychological assessments and clinical semiology, and as a clue for discovering pathophysiological mechanisms between brain glucose metabolism and cognitive decline and vascular risk factors in AD dementia.

Cognitive impairment arising from neuronopathies and neuronal apoptosis is a key manifestation of the AD spectrum. We explored the potential association between glucose metabolism indirectly reflecting neuronal activities and cognitive conditions encompassing comprehensive cognition scores and single-domain cognitive assessments. Extensive population-based studies have consistently shown a positive correlation between ^18^F-FDG regional uptake and cognitive function measured by the MMSE in AD profiles.^10,23,24^ Specifically, Khosravi et al.^24^ suggested that MMSE was positively correlated with the temporal lobe in both MCI and AD populations. However. our current findings indicate a less significant association between MMSE scores and glucose uptake in MCI participants. The temporal lobe is implicated in human language comprehension, emotion, memory, and facial recognition, particularly the medial temporal lobe, which is a key factor in the formation of “declarative memory”. This might be the explanation that MMSE scale used to assess memory and multiple cognitive functions is closely related to temporal lobe metabolism. For another widely used and extensively studied questionnaire-based neuropsychological assessment, Jing et al.^25^ reported a finding that the degree of ^18^F-FDG metabolism in brain functional areas like the temporal and parietal lobes was positively correlated with the MoCA total scores in AD patients, which is consistent with our results from the AD-populated analysis. Overall, our analysis based on the AD population revealed positive correlations similar to those reported in previous studies, but no similar association was observed between metabolism and cognitive scores in the MCI population. Notably, the assessment of single-domain cognition yielded different results, with significant associations observed only in the MCI group. MCI represents an intermediate state between normal aging and dementia, characterized by objective evidence of decline in one or more cognitive domains, distinct from the progressive and global cognitive impairment seen in AD. Therefore, it is speculated that this discrepancy in disease severity might indicate patterns and routes of neuropathological progression as demonstrated by neuroimaging with ^18^F-FDG PET, and ^18^F-FDG PET might be useful for predicting cognitivedecline.

In addition to the well-studied cognitive scales, we looked into the less researched cognitive correlation scales used in the diagnostic and differential process of AD, such as ADL, GDS, HIS, and HAMA. We observed that ADL, which assesses an individual’s ability to perform daily living, was negatively related to the metabolism in the occipital and frontal lobes in AD patients. Moreover, the GDS, a measure of depressive symptom severity in older adults, showed a negative correlation with metabolism in the hippocampus and parietal lobe in AD patients. The superior frontal gyrus is primarily responsible for processing information related to exercise. The hippocampus interacting with the amygdala is involved in processing emotional responses. An increase in ADL and GDS scores suggests a more advanced disease stage, therefore, inhibition of neural function and metabolism in the corresponding brain regions is more evident. Our study is a cut-and-try exploration of less commonly used scales and have attained outcomes of significant substance. Further research is warranted to ascertain the feasibility and applicability of these findings.

It is documented that cardiovascular risk factors and an unhealthy lifestyle are closely linked to the risk and progression of dementia.^1,12^ A current study targeting Aβ deposition indicated that diabetes, hypertension, and hypercholesterolemia contribute to the Aβ pathology of AD in a general population of dementia-free participants.^12^ In our study, we discovered statistically significant evidence that physical condition (BMI) and serologic markers related to diabetes, hypertension, and hypercholesterolemia were associated with reginal glucose metabolism in MCI patients, as visualized by ^18^F-FDG PET. However, this association was not observed in AD patients. Previous research emphasized that diabetes and AD share numerous common cellular and molecular pathways, including impaired insulin signaling, chronic hyperglycemia, and inflammatory effects. Among these, insulin resistance stimulating *γ*-secretase activity is likely an etiological factor due to its critical role in Aβ accumulation.^26^ On the contrary, Roberts et al.^27^ conducted a study investigating the associations of type 2 diabetes with amyloid accumulation and brain hypometabolism among 749 nondemented participants. They discovered that diabetes and elevated HbA1c levels are linked to brain hypometabolism but not to amyloid deposition. An animal experiment showed that under diet-induced obesity conditions, improvement in cognitive function and alleviation of the hypermetabolic state of the brain induced by free fatty acid receptor 3 (FFR3) ablation in a mouse model of Tg2576 commonly used to mimic the pathological features of AD dementia was observed, providing novel insights into the mechanisms linking obesity, metabolic disorders, and cognitive function.^28^ Otherwise, animal models provided that hypertension can induce AD-like pathological changes in the mouse cerebral cortex, with the biological plausibility of this association being supported by the concept that plaque formation results from long-term vascular insults.^14^ Extensive animal or human studies have shed light on the role of various risk factors in promoting the development of non-AD states to AD-like pathological states. Our study explored the effects of existing vascular risk factors on brain metabolism in the context of MCI and AD and found meaningful results in the MCI-only group, suggesting that vascular-related risk factors may not only increase the likelihood of developing AD but also play a role in the progression from MCI to AD. Due to the limited nature of current research, we have not been able to expound how serology linked to vascular risk factors affects brain metabolism, but provided a foundation for future studies to investigate whether controlling and reversing risk factors during the MCI stage can delay disease progression, as well as to assess and monitor its effect using ^18^F-FDG PET as a tool.

In our study, both visual interpretation and voxel-based quantitative methods were employed to differentiate dementia subtypes, yielding meaningful results, particularly for the identification of FTD based on quantitative analysis. Over the past few decades, the application of ^18^F-FDG PET in neurodegenerative disorders has been described widely in the literature. The classic findings in AD dementia include hypometabolism in the parietal and temporal associated cortices, the posterior cingulate cortex, and the precuneus. In contrast, FTD is primarily characterized by reduced metabolism in the frontal and anterior temporal cortices.^8^ Our voxel-based analysis corroborated these patterns. Nevertheless, we did not show hypometabolism in the temporal lobe specific to either disease in quantitative analysis, which may be related to the involvement of the temporal lobes in both conditions. Genetic and pathological studies revealed that both AD and FTD involve tau protein burden,^29^ which complicates the use of tau-PET for disease management. In such cases, the incorporation of ^18^F-FDG PET imaging proves beneficial. When comparing AD profiles, our quantitative analysis revealed minor significant differences in metabolism among MCI and AD subtypes (except for mild AD). But an updated review of the recent advances (2017–2022) conducted by Cotta Ramusino et al.^30^ suggested that molecular imaging techniques have unstable performance in predicting the progression from MCI to AD dementia. For ^18^F-FDG PET, sensitivities varied from 43% to 100%, and specificities ranged from 63% to 94%. For amyloid-PET, the respective ranges were 64% to 94% for sensitivities and 48% to 93% for specificities. It is noted that an integrated imaging approach that combines MR anatomical imaging and PET molecular imaging has shown promise in improving the classification accuracy of MCI and AD,^31^ which may be a worthy direction for futureresearch.

Several limitations in the current study warrant attention. Firstly, due to the retrospective nature of the study, longitudinal data on changes in glucose metabolism and serological markers were unavailable, which may have obscured the relationships among the observed variations. It has been reported that the *APO*E gene is associated with the Aβ burden in AD.^12^ Because of the restrictions of financial situation and clinical practice, genetic testing was not conducted for all AD patients, precluding a genetic influence analysis on glucose metabolism in this study. Lastly, the sample size of our research is relatively small. Further studies with larger cohorts are needed to substantiate our findings.

### Conclusion

In conclusion, this study outlines the utility of molecular imaging with ^18^F-FDG PET in differentiating AD dementia and staging its progression. It also suggests that cognitive function scores are associated with glucose metabolism in specific brain regions of AD, while single-domain cognitive performance and vascular risk factors are associated with metabolic accumulation of MCI specific brain regions. ^18^F-FDG PET might be a promising option for predicting cognitive decline and serve as a visual platform for studying the underlying mechanism by which vascular risk factors contribute to the transition from MCI to AD.

## AUTHOR CONTRIBUTIONS

Min Xiong (Conceptualization; Formal analysis; Investigation; Methodology; Project administration; Writing – original draft); Hongji You (Data curation; Methodology; Project administration; Software; Validation); Wang Liao (Data curation; Project administration; Resources; Supervision; Validation); Yingren Mai (Data curation; Project administration; Resources; Supervision; Validation); Xiaoming Luo (Resources; Software); Yipei Liu (Resources; Software); Sheng-Nan Jiang (Supervision; Validation; Writing – review & editing).

## Data Availability

The data supporting the findings of this study are available on request from the corresponding author.
